# Long-term Success of Partial Ganglionated Plexus Ablation in a Patient with Tachycardia–bradycardia Syndrome and Syncope: Whom and How?

**DOI:** 10.19102/icrm.2021.121006

**Published:** 2021-10-15

**Keywords:** Ablation, cardioneuroablation ganglionated plexi, syncope, tachy–brady syndrome

## Drs. Yalin, Altinsoy, Soysal, and Aksu comment

The cardiac autonomic system holds a key role in the development and treatment of various cardiac arrhythmias. Sympathetic overactivity or imbalance in favor of the sympathetic system may cause inappropriate sinus tachycardia and some forms of ventricular tachycardia.^1^ Moreover, vasovagal syncope, functional atrioventricular (AV) block, and some forms of sinus node dysfunction are several results of an imbalance in the cardiac autonomic system or parasympathetic overactivity.^2^ Thus, targeting the autonomic nervous system may solve these kinds of arrhythmic disorders. Surgical sympathectomy and percutaneous sympathetic cardiac denervation are well-known treatment strategies of malignant refractory ventricular tachycardias.^3^ In recent years, catheter-based autonomic neuromodulation or ablation of ganglionated plexi (GPs) or cardioneuroablation (CNA) has emerged as an important novel therapy method for parasympathetic system–related conditions. The concept of GPs includes that the intrinsic cardiac autonomic nervous system consists of grouping epicardial ganglia.^4^ The technique was first introduced by Dr. Pachon and colleagues for vasovagal syncope, functional AV block, and vagal sinus bradycardia.^5^ Our group verified and expanded the experience on CNA in a similar cohort.^6^

In this issue of *The Journal of Innovations in Cardiac Rhythm Management*, Dr. Gorev and colleagues offer a case report of a patient who presented with consecutive syncope and palpitation attacks, which were related to intermittent AV block and paroxysmal atrial fibrillation (AF), and who was treated with ablation of the GPs in addition to pulmonary vein (PV) isolation (PVI).^7^ The authors obtained satisfying results without any recurrence of palpitations or syncope during a three-year follow-up period. It is noteworthy, however, that ablation of the GPs requires many considerations and prudence due to the complexity of sinoatrial and AV nodal vagal innervation.^4,8^ In addition, the patient had a mixed pathology consisting of AF and atrial flutter episodes as well as pauses lasting up to 13 seconds with high-degree AV block followed by sinus node slowing during the night. Sinus node dysfunction and AV block are usually associated with intrinsic fibrosis of the conduction system, while, in a selected group of patients, excessive vagal tone may be the only cause. Regarding the differential diagnosis of AV block, vagal or functional AV block, intrinsic or structural AV block, and extrinsic idiopathic AV block should be considered.^9^ The vagally mediated form can be distinguished by AV block preceded by sinus node slowing, and sinus rhythm during AV block is slow and unstable. Progressive P–R prolongation before a high-degree AV block episode as documented in the present case may also be noticeable. Therefore, continuous Holter tracing examination of the AV block before, during, and after the episode is essential. If these features are not present, an increase in sinus rate and the resolution of AV block with atropine bolus may be useful.^10^ On the contrary, the presence of a low-voltage area supports the possibility of intrinsic fibrosis of the conduction system by suggesting structural involvement.

The observation of an increase in sinus rate after PVI in some individuals may give rise to the idea of resolution of AV block following PVI. The great majority of the epicardial ganglia of GPs are located adjacent to the left atrium and PV junctions, and wide antral ablation around PVs may result in an increase in sinus rate due to some vagal denervation.^11^ However, these autonomic effects are not universal, and the reasons behind the variability in this observation are still unclear. In a recently published study, anatomical overlap of GPs with the traditional PVI lesion set was investigated by our group.^12^ In 38 patients with vagally mediated arrhythmias, design PVI lines located 1 cm away from the PV ostia were created by a blinded observer on left atrial electroanatomic maps (sans the ablation tags). The co-localization and overlap between the design lines were compared to electroanatomically located GPs, and 11.7 ± 7 (35.5% ± 17.0%) of the total 31.6 ± 10 GP ablation sites per patient were found to overlap with the design PVI lines, with the degree of overlap being greater for the right-sided GPs. The degree of GP–PV overlap varied as follows: one PV in five (13.2%) patients, two PVs in 15 (39.2%) patients, three PVs in 16 (42.1%) patients, and all four PVs in two (5.3%) patients. It should be noted that, if GP ablation is needed, PVI itself is not usually sufficient to warrant parasympathetic denervation in the majority of patients.

In the present case, the authors performed additional empirical anatomical ablation in the right superior and left superior GPs following the completion of PVI and the cavotricuspid isthmus line. However, the anatomical location and the number of GPs identified during an electrophysiological study may vary significantly among patients. To perform a more specific and restricted ablation without compromising the success, different groups have suggested different methods, like high-frequency stimulation and electrogram analysis, taking into account these anatomical variations during electrophysiological study.^2,4^ With this aim, we defined a fragmented electrogram–guided GP ablation strategy and compared this technique with a hybrid approach in which a combination of high-frequency stimulation, spectral analysis, and additional anatomical ablation was used.^13^ Of note, the median event-free survival was comparable between the groups. The main advantage of this technique is the opportunity to localize GPs without using any additional equipment during electrophysiological study.

Previously, only the second parasympathetic neuron existence was described in the GPs, whereas it is now well known that the epicardial ganglia of GPs contain both efferent parasympathetic and sympathetic neuronal somas and presumably local circuit neurons/interneurons.^14^ Considering a similar distribution of sympathetic innervation, the achievement of similar and durable denervation on the sympathetic system might be possible after GP ablation. Our group demonstrated a significant and durable shortening of the corrected QT interval (QTc) after GP ablation in patients with a normal QTc interval and long QT syndrome.^15,16^ Shortening of the QTc interval was attributed to the additional sympatholytic effect of GP ablation. In the subject case, tachycardia–bradycardia (tachy–brady) syndrome and ablation of left superior and right superior GPs with possible sympathetic fiber damage in addition to parasympathetic denervation may be another reason for the treatment of inappropriate tachycardia episodes.

On the basis of human data, the right superior GP mainly supplies epicardial nerves to the sinoatrial nodal neural network.^17^ Compatible with this anatomical description, Hu et al.^18^ demonstrated that ablation of the right superior GP region promotes a significant increase in the sinus rate. Despite the results of physiological experiments, successive anatomical attempts to determine nerves that coursed toward the AV node failed as those endocardial neural nerves are especially tiny.^19^ However, our previous experience reinforces that the inferior vena cava–left atrial fat pad region or the posteromedial left atrial GP might be the ablation target for vagal denervation of the AV node.^10,20^ In the present case, additional ablation of the posteromedial left atrial GP would have increased the patency of AV conduction.

There is uncertainty regarding what endpoints to employ for individual GP ablation and for the overall GP ablation procedure. In the subject case, the authors have no clear endpoint for GP ablation. In our current approach, elimination of targeted fragmented atrial electrograms and the abolition of vagal response in a previously vagal response–positive site are considered markers of successful GP ablation.^14–16^ The response to intravenous atropine pre- and postablation might be used as a procedural endpoint, and the goal of the ablation procedure is to eliminate this atropine response. Pachon et al.^21^ used extracardiac vagal stimulation by placing a catheter in the internal jugular vein, allowing an elegant evaluation of vagal denervation at each step of the procedure. However, this approach has not been replicated by other groups due to its contribution to the complexity and duration of the procedure. An attempt to obtain electrocardiogram documentation by an implantable loop recorder might increase the diagnostic accuracy of follow-up in future studies.^22^

The present case conduces to increase knowledge about the GP ablation technique. We believe that growing evidence of the safety and efficacy of GP ablation with a relatively long follow-up duration and reproducibility of the ablation technique by different operators will expand its usage worldwide.

Kivanc Yalin, md,^1^ Meltem Altinsoy, md,^2^ Ali Ugur Soysal, md,^1^ and Tolga Aksu, md (aksutolga@gmail.com)^3^

^1^Department of Cardiology, Cerrahpasa Faculty of Medicine, Istanbul University—Cerrahpasa, Istanbul, Turkey

^2^Department of Cardiology, Diskapi Education and Research Hospital, University of Health Sciences, Ankara, Turkey

^3^Department of Cardiology, Faculty of Medicine, Yeditepe University, Istanbul, Turkey

The authors report no conflicts of interest for the published content.

References1.MengLShivkumarKAjijolaOAutonomic regulation and ventricular arrhythmiasCurr Treat Options Cardiovasc Med201820538[CrossRef][PubMed]2962787110.1007/s11936-018-0633-z2.AksuTGulerTEYalinKMutluerFOOzcanKSCalòLCatheter ablation of bradyarrhythmia: from the beginning to the futureAm J Med Sci20183553252265[CrossRef][PubMed]2954992810.1016/j.amjms.2017.11.0163.YalinKLiosisSPaladeECardiac sympathetic denervation in patients with nonischemic cardiomyopathy and refractory ventricular arrhythmias: a single-center experienceClin Res Cardiol202111012128[CrossRef][PubMed]3232873510.1007/s00392-020-01643-84.AksuTGopinathannairRGuptaDPauzaDHIntrinsic cardiac autonomic nervous system: what do clinical electrophysiologists need to know about the “heart brain”?J Cardiovasc Electrophysiol202132617371747[CrossRef][PubMed]3392871010.1111/jce.150585.PachonJCPachonEIPachonJC“Cardioneuroablation”—new treatment for neurocardiogenic syncope, functional AV block and sinus dysfunction using catheter RF-ablationEuropace200571113[CrossRef][PubMed]10.1016/j.eupc.2004.10.003156709606.AksuTGolcukEYalinKGulerTEErdenISimplified cardioneuroablation in the treatment of reflex syncope, functional AV block, and sinus node dysfunctionPacing Clin Electrophysiol20163914253[CrossRef][PubMed]2641127110.1111/pace.127567.GorevMVNardaiaSGSergeevaOAVasilievaEYShpektorAVRzaevFGLong-term success of cardioneuroablation in a patient with tachy–brady syndrome and syncopeJ Innov Cardiac Rhythm Manage202112104715471910.19102/icrm.2021.121001PMC8545437347125068.AksuTGuptaDPauzaDHAnatomy and physiology of intrinsic cardiac autonomic nervous system: Da Vinci Anatomy Card #2JACC Case Rep202134625629[CrossRef][PubMed]3431759010.1016/j.jaccas.2021.02.018PMC83027929.AksuTGulerTEBozyelSYalinKPotential usage of cardioneuroablation in vagally mediated functional atrioventricular blockSAGE Open Med201972050312119836308. [CrossRef][PubMed]10.1177/2050312119836308PMC64215943090655110.AksuTGülerTEYalınKStep-by-step cardioneuroablation approach in two patients with functional atrioventricular blockBalkan Med J2019366301310[CrossRef][PubMed]3164843510.4274/balkanmedj.galenos.2019.2019.9.47PMC683515711.ArmourJAMurphyDAYuanBXMacdonaldSHopkinsDAGross and microscopic anatomy of the human intrinsic cardiac nervous systemAnat Rec19972472289298[CrossRef][PubMed]902600810.1002/(SICI)1097-0185(199702)247:2<289::AID-AR15>3.0.CO;2-L12.AksuTYalinKBozyelSGopinathannairRGuptaDThe anatomical basis behind the neuromodulation effects associated with pulmonary vein isolationJ Cardiovasc Electrophysiol202132617331736[CrossRef][PubMed]3384439510.1111/jce.1503813.AksuTGulerTEMutluerFOBozyelSGolcukSEYalinKElectroanatomic-mapping-guided cardioneuroablation versus combined approach for vasovagal syncope: a cross-sectional observational studyJ Interv Card Electrophysiol2019542177188[CrossRef][PubMed]3005482810.1007/s10840-018-0421-414.CrickSJAndersonRHHoSYSheppardMNLocalization and quantitation of autonomic innervation in the porcine heart II: endocardium, myocardium and epicardiumJ Anat1999195Pt 3359373[CrossRef][PubMed]1058085110.1046/j.1469-7580.1999.19530359.xPMC146800515.AksuTGulerTEBozyelSMedium-term results of cardioneuroablation for clinical bradyarrhythmias and vasovagal syncope: effects on QT interval and heart rateJ Interv Card Electrophysiol20216015768[CrossRef][PubMed]3203461110.1007/s10840-020-00704-216.AksuTGulerTEBozyelSYalinKGopinathannairRPotential therapeutic effects of electrogram-guided cardioneuroablation in long QT syndrome: case seriesJ Interv Card Electrophysiol2021612385393[CrossRef][PubMed]3270012910.1007/s10840-020-00831-w17.PauzieneNPauzaDHStropusRMorphology of human intracardiac nerves: an electron microscope studyJ Anat2000197Pt 3437459[CrossRef][PubMed]1111762910.1046/j.1469-7580.2000.19730437.xPMC146814418.HuFZhengLLiangERight anterior ganglionated plexus: the primary target of cardioneuroablation?Heart Rhythm2019161015451551[CrossRef][PubMed]3133018710.1016/j.hrthm.2019.07.01819.SaburkinaIRysevaiteKPauzieneNEpicardial neural ganglionated plexus of ovine heart: anatomic basis for experimental cardiac electrophysiology and nerve protective cardiac surgeryHeart Rhythm201077942950[CrossRef][PubMed]2019711810.1016/j.hrthm.2010.02.036PMC302518320.AksuTGulerTEBozyelSYalinKSelective vagal innervation principles of ganglionated plexi: step-by-step cardioneuroablation in a patient with vasovagal syncopeJ Interv Card Electrophysiol2021603453458[CrossRef][PubMed]3239410410.1007/s10840-020-00757-321.Pachon-MJCPachon-MEIPachonCTCLong-term evaluation of the vagal denervation by cardioneuroablation using holter and heart rate variabilityCirc Arrhythm Electrophysiol20201312e008703[CrossRef][PubMed]3319848610.1161/CIRCEP.120.00870322.AksuTGulerTESaygıSYalinKUsage of implantable loop recorder to evaluate absolute effectiveness of cardioneuroablationJ Cardiovasc Electrophysiol2019301229862987[CrossRef][PubMed]3162637810.1111/jce.14243

## Dr. Gopinathannair discusses

In this well-written case report, Gorev et al. report on a 48-year-old man with paroxysmal palpitations and recurrent syncope.^1^ Cardiac monitoring revealed AF with rapid ventricular response as well as typical atrial flutter along with long asystolic pauses of 13 seconds during AF due to AV block. There were also episodes of vagally mediated AV block during night hours. Use of propafenone was ineffective in suppressing AF and worsened bradycardia and symptomatic AV block. The patient refused pacemaker implantation. An atropine challenge resulted in the resumption of 1:1 conduction and shortening of the P–R interval, suggesting a vagally mediated mechanism for the AV block.

The authors performed an ablation of the right superior and left superior GPs, guided by fractionated electrograms during sinus rhythm,^2^ along with bilateral PVI and cavotricuspid isthmus ablation. After GP ablation, especially of the right superior GP, the sinus rate accelerated from 60 to 75 bpm, and the sinus rate at the end of the procedure was 68 to 70 bpm. At three years of follow-up, the patient was in sinus rhythm with intact AV conduction and was free from any recurrent syncope.

The report shows the impact of CNA in preventing syncope secondary to functional, vagally mediated AV block. I commend the authors for recognizing the clinical situation and performing appropriate testing to determine the functional nature of the AV block. However, I think the characterization by the authors that this is a case of tachy–brady syndrome may not be accurate. Unless there have been other episodes where there were conversion sinus pauses, the tracings shown and described in the text are consistent with AV block during AF and vagally mediated bradycardia during nocturnal hours. I suggest that the authors thus modify their title to “Cardioneuroablation in a patient with functional/vagally mediated AV block.” Typically, posteromedial left GP (PMLGP) ablation is important in CNA for functional AV block. In a recent study, in 12 of 15 (80%) patients with persistent AV block, PMLGP ablation resulted in the return of 1:1 conduction, whereas only three out of 15 patients showed resumed 1:1 conduction after right superior GP ablation.^3^ Also, bi-atrial ablation has been shown to be better than right atrial ablation alone.^4^ However, in this case, the authors achieved acute success with only right superior GP ablation, without superior vena cava–aorta GP and/or PMLGP ablation.

The acute and medium-term effects of CNA for functional AV block were recently published.^3^ CNA resulted in the acute reversal of AV block in 31 patients with functional AV block and complete abolition of the atropine response in 30 (96.7%) patients. Over a 19.3- ± 15-month follow-up period, recurrent AV block episodes were observed in two (6.7%) of the 30 patients, and three (9.6%) patients required pacemaker implantation. No major complications associated with CNA were noted.^3^

An important caveat is to make sure that the AV block is functional, and, in this report, the authors evaluated it appropriately using the atropine challenge test. In the study from Aksu et al., out of 241 consecutive patients presenting with symptomatic AV block, only 31 (12.9%) had functional or vagally mediated AV block.^3^ CNA offers a fresh lease of life to these patients without having to have a permanent pacemaker and the long-term issues associated with it. However, randomized trials with long-term follow-up are warranted to establish this as a mainstream therapy.

Rakesh Gopinathannair, md, ma, faha, facc, fhrs (rakesh.gopinathannair@louisville.edu)^1,2^

^1^Kansas City Heart Rhythm Institute, Overland Park, KS, USA

^2^University of Missouri—Columbia, Columbia, MO, USA

Dr. Gopinathannair reports no conflicts of interest for the published content.

References1.GorevMVNardaiaSGSergeevaOAVasilievaEYShpektorAVRzaevFGLong-term success of cardioneuroablation in a patient with tachy–brady syndrome and syncopeJ Innov Cardiac Rhythm Manage202112104715471910.19102/icrm.2021.121001PMC8545437347125062.AksuTErdem GulerTGopinathannairRLetter by Aksu et al. regarding article, “Relation of fractionated atrial potentials with the vagal innervation evaluated by extracardiac vagal stimulation during cardioneuroablation”Circ Arrhythm Electrophysiol2020138e008595[CrossRef][PubMed]3280987910.1161/CIRCEP.120.0085953.AksuTGopinathannairRBozyelSYalinKGuptaDCardioneuroablation for treatment of atrioventricular blockCirc Arrhythm Electrophysiol2021149e010018[CrossRef][PubMed]3446512210.1161/CIRCEP.121.0100184.AksuTGulerTEBozyelSMedium-term results of cardioneuroablation for clinical bradyarrhythmias and vasovagal syncope: effects on QT interval and heart rateJ Interv Card Electrophysiol20216015768[CrossRef][PubMed]3203461110.1007/s10840-020-00704-2

## Drs. Braunstein and Cheung examine

Gorev et al. describe a case of AF and flutter causing palpitations, with concomitant syncope likely due to bradycardia in a 48-year-old man who was successfully treated with PVI, CNA, and cavotricuspid isthmus ablation.^1^ This case highlights an emerging role for CNA for the treatment of syncope in select patients due to bradycardia. The causes of bradyarrhythmias are diverse, with the most common being primary conduction disease, which can be due to degenerative fibrosis, inflammatory and infiltrative conditions, infections, or ischemia. Secondary bradyarrhythmias due to increased efferent cardiac vagal inputs can occur in the setting of heightened systemic vagal tone or central or peripheral baroreceptor reflex abnormalities.^2^ Regardless of the cause, clinical presentations of syncope caused by sinus arrest or paroxysmal AV block can be seen, and current guidelines recommend the treatment for these disorders with permanent pacing after reversible causes such as drug toxicity, Lyme carditis, and sleep apnea are excluded.^3^ However, severe cardioinhibitory syncope can affect younger patients, where the long-term complications of cardiac implantable electronic devices may be increased, and therefore alternative treatments should be considered.

In this case report by Gorev et al., the patient had paroxysmal AF, atrial flutter, profound ventricular pauses, and a history of syncope. Most commonly, the association between paroxysmal AF and syncope is due to the presence of prolonged post-conversion pauses. In such cases, catheter ablation of AF with PVI is sufficient for preventing recurrent bradycardia by eliminating the AF and its associated conversion pauses altogether. However, in this case, the patient had complete AV block while in sinus rhythm, suggesting that the syncope was not due to conversion pauses. He had prolonged ventricular pauses during AF of up to 12.9 seconds, which would support the diagnosis of a paroxysmal AV block. Moreover, sinus slowing was seen during another episode of complete AV block, which would suggest that it was vagotonic in mechanism. Therefore, these findings would suggest that cardioinhibitory syncope may have been the dominant etiology of the patient’s syncope instead of the usual post-conversion pauses typically associated with paroxysmal AF. The authors do not report whether any of the episodes of AV block during the Holter recordings were symptomatic, which would have helped to clarify the precise mechanism of syncope.

Current recommended treatments for vasovagal syncope include lifestyle modification (avoiding triggering factors, increased fluid and salt intake, compression stockings, and isometric counterpressure maneuvers) and medications (selective serotonin reuptake inhibitors, β-blockers, midodrine, fludrocortisone, and ivabradine).^4^ However, failure of these treatments is common, and permanent pacing is often pursued in patients with severe cardioinhibitory syncope with documented sinus arrest or AV block, even in the face of conflicting evidence of the utility of permanent pacing in clinical trials.^5^

CNA has emerged as an alternative therapy for cardioinhibitory syncope, which typically consists of targeting GPs in the left and right atria through energy delivery with an ablation catheter.^6^ Identification of the location of the GPs is a critical part of CNA. Common GP sites include the posterior wall, roof and anterosuperior areas of the left atrium near both the PV–atrial junctions, the ligament of Marshall, the superior vena cava, the coronary sinus ostium, and the superoposterior right atrium.^7^ As the GPs are epicardial structures that reside in the pericardial fat, epicardial GP ablation during thoracoscopic surgical AF ablation has also been described.^8^ Multiple techniques for GP localization have been discussed. High-frequency stimulation at 20 Hz, with the goal of elucidating a vagal cardiac response, has been used to determine the locations of vagal innervation sies.^9^ Pachon et al. reported using spectral analysis of endocardial electrograms to identify GPs.^10^ Aksu et al. reported the presence of fractionated electrograms at common GP sites.^11^

Owing to the location of the left atrial GPs around the PVs, GP ablation is often performed, intentionally or not, as part of PVI for the treatment of AF. The vagolytic effects of PVI have been extensively documented and are thought to be one of the mechanisms by which PVI can successfully eliminate AF. Additional GP modification targeting left atrial GPs during AF ablation is often pursued for this reason.^12^ Therefore, in this case report by Gorev et al., the presence of concurrent AF and vasovagal syncope in the patient permitted adjunctive CNA at the time of PVI. The authors did not employ high-frequency stimulation or electrogram analysis to localize the left atrial GPs and simply used a purely anatomic approach by delivering empiric ablation at the left atrial roof near the left superior PV and the anterior wall by the ostium of the right superior PV. The posterior left atrial GPs were not targeted. The authors found evidence of good short-term vagolytic effect after GP ablation, as manifested by an increase in resting heart rate coupled with decreases in the P–R interval, AV nodal effective refractory period, and sinus node recovery time. The patient remained free from syncope after several years of follow-up and avoided cardiac implantable electronic device implantation, underscoring the utility of this treatment.

Currently, the evidence to support the use of CNA as the first-line treatment for vasovagal syncope, sinus node dysfunction, and AV block is limited. While several small trials have shown promise for this treatment in properly selected patients, larger studies with optimization of patient selection and standardization of procedural techniques are needed.^12–14^ Nonetheless, in patients with AF and vagotonic AV block for whom PVI is already required, adjunctive CNA appears to be safe and potentially effective at preventing the need for permanent pacing, as underscored nicely in the case report by Gorev et al.

Eric D. Braunstein, md^1^ and Jim W. Cheung, md (jac9029@med.cornell.edu)^1^

^1^Division of Cardiology, Department of Medicine, New York Presbyterian Hospital, Weill Cornell Medicine, New York, NY, USA

Dr. Cheung has received consulting and speaking fees from Abbott, Biotronik, and Boston Scientific and fellowship grant support from Abbott, Biosense, Biotronik, Boston Scientific, and Medtronic. Dr. Braunstein reports no conflicts of interest for the published content.

References1.GorevMVNardaiaSGSergeevaOAVasilievaEYShpektorAVRzaevFGLong-term success of cardioneuroablation in a patient with tachy–brady syndrome and syncopeJ Innov Cardiac Rhythm Manage202112104715471910.19102/icrm.2021.121001PMC8545437347125062.Ben-MenachemEReveszDSimonBJSilbersteinSSurgically implanted and non-invasive vagus nerve stimulation: a review of efficacy, safety and tolerabilityEur J Neurol201522912601268[CrossRef][PubMed]2561417910.1111/ene.12629PMC50240453.KusumotoFMSchoenfeldMHBarrettC2018 ACC/AHA/HRS guideline on the evaluation and management of patients with bradycardia and cardiac conduction delay: executive summary: a report of the American College of Cardiology/American Heart Association Task Force on Clinical Practice Guidelines, and the Heart Rhythm SocietyCirculation20191408e333e381[CrossRef][PubMed]3058677110.1161/CIR.00000000000006274.ShenWKSheldonRSBendittDG2017 ACC/AHA/HRS guideline for the evaluation and management of patients with syncope: a report of the American College of Cardiology/American Heart Association Task Force on Clinical Practice Guidelines and the Heart Rhythm SocietyJ Am Coll Cardiol2017705e39e110[CrossRef][PubMed]2828622110.1016/j.jacc.2017.03.0035.GopinathannairRSalgadoBCOlshanskyBPacing for vasovagal syncopeArrhythmia Electrophysiol Rev20187295[PubMed]10.15420/aer.2018.22.2PMC6020179299676816.PachonMJCPachonMEICunha PachonMZLoboTJPachonMJCSantillanaPTGCatheter ablation of severe neurally meditated reflex (neurocardiogenic or vasovagal) syncope: cardioneuroablation long-term resultsEP Eur201113912311242[CrossRef][PubMed]10.1093/europace/eur163217122767.AksuTGopinathannairRGuptaDPauzaDHIntrinsic cardiac autonomic nervous system: what do clinical electrophysiologists need to know about the “heart brain”?J Cardiovasc Electrophysiol202132617371747[CrossRef][PubMed]3392871010.1111/jce.150588.HaldarSInvestigators on behalf of the C-AKhanHRCatheter ablation vs. thoracoscopic surgical ablation in long-standing persistent atrial fibrillation: CASA-AF randomized controlled trialEur Heart J2020414744714480[CrossRef][PubMed]3286041410.1093/eurheartj/ehaa658PMC77676349.PokushalovERomanovAArtyomenkoSGanglionated plexi ablation directed by high-frequency stimulation and complex fractionated atrial electrograms for paroxysmal atrial fibrillationPacing Clin Electrophysiol2012357776784[CrossRef][PubMed]2248621510.1111/j.1540-8159.2012.03392.x10.PachonMJCPachonMEIPachonMJC“Cardioneuroablation”—new treatment for neurocardiogenic syncope, functional AV block and sinus dysfunction using catheter RF-ablationEP Eur200571113[CrossRef][PubMed]10.1016/j.eupc.2004.10.0031567096011.AksuTGolcukEYalinKGulerTEErdenISimplified cardioneuroablation in the treatment of reflex syncope, functional AV block, and sinus node dysfunctionPacing Clin Electrophysiol20163914253[CrossRef][PubMed]2641127110.1111/pace.1275612.AvazzadehSMcBrideSO’BrienBGanglionated plexi ablation for the treatment of atrial fibrillationJ Clin Med20209103081[CrossRef][PubMed]10.3390/jcm9103081PMC75987053298782013.YaoYShiRWongTEndocardial autonomic denervation of the left atrium to treat vasovagal syncopeCirc Arrhythmia Electrophysiol201252279286[CrossRef][PubMed]10.1161/CIRCEP.111.9664652227548514.AksuTGopinathannairRBozyelSYalinKGuptaDCardioneuroablation for treatment of atrioventricular blockCirc Arrhythmia Electrophysiol2021149e010018[CrossRef][PubMed]10.1161/CIRCEP.121.01001834465122
